# The optimum inhibitory effects of Alpha Interferon and Cisplatin in colon cancer, a comparative *in vitro* study

**DOI:** 10.25122/jml-2021-0336

**Published:** 2022-02

**Authors:** Arafat Muttar, Ihab Ahmed, Huda Hameed

**Affiliations:** 1. Ministry of Higher Education and Scientific Research, Baghdad, Iraq; 2. Department of Pharmacology, College of Pharmacy, Al-Bayan University, Baghdad, Iraq

**Keywords:** colon cancer, *in vitro*, cisplatin

## Abstract

Cisplatin is well known as a potent anti-cancer agent against colon cancer. However, alpha interferons are also widely used for cancer suppression. This *in vitro* study was designed to investigate and compare the cancer suppression function of alpha interferon in colon cancer with Cisplatin. The analysis used a human SW 480 cancer cell line with RPMI-1630 culture media. Six dilutions of interferon (2.5 μg/ml, 1.25 μg/ml, 0.562 μg/ml, 0.286 μg/ml, 0.143 μg/ml, and 0.057 μg/ml) and six dilutions of cisplatin (100 μg/ml, 50 μg/ml, 25 μg/ml, 6.25 μg/ml, and 3.125) were used at 24, 48 and 72 hours along with the presence of control groups. Following this, results were observed by ELISA plate reader, and percentage inhibition was calculated using ANOVA analysis. The interferon and cisplatin percentage of inhibition was comparable with higher inhibition rates observed with alpha interferon. The statistical analysis showed that the maximum inhibition was observed at a 0.143 μg/ml interferon concentration when exposed for 48 to 72 hours. This *in vitro* analysis demonstrated the anti-cancer activity of alpha interferon and its advanced inhibitory activity compared to Cisplatin.

## Introduction

Cancer stands as the most common cause of death with a significant effect on patients’ quality of life. Overall, the incidence and mortality of cancer are rapidly increasing with the increasing population. Colon cancer is the 5^th^ most common cancer, with 1,148,515 new cases and 576,858 deaths reported in 2020 worldwide [1, 2]. Despite several treatment options, we struggle to reduce mortality in diseased people [2, 3]. In most cases, surgical interventions are effective in colon cancer, but almost 50% of patients suffer from relapse with metastasis. Adjuvant therapy and surgery are essential to prevent this relapse [3, 4]. Cisplatin, a platinum-based chemical compound, is one of the most potent and effective anti-cancer agents used in various malignancies [5]. It is often used in colon cancer too. It works by entering the cancerous cells and damaging their DNA [6]. Cisplatin is known to cause cell death and stimulate CD-95 mediated apoptosis [7]. Despite its successful treatment results in other cancers, its high toxicity and resistance in colorectal cancer limit its usage in clinical settings [3, 4]. It has been observed that chemotherapy with cisplatin results in treatment failure and tumor recurrence in colon cancer [5]. Alpha interferons are naturally occurring chemicals in our body but are also extensively used to treat viral ailments. These are usually present in small amounts and inhibit protein production in cancerous cells, resulting in an increased cell cycle duration and modulated oncogene expression. Although the exact mechanism of action of interferons is unknown [7], it is believed that alpha interferon works through multiple methods [8]. It is considered that the intrinsic function of interferon is tumor suppression by regulating the expression of various genes that modulate the tumor cell growth, proliferation, migration, and antigen expression. Originally, these were known for suppressing viral replication, but now their use is extended for cancer suppression [9]. Alpha interferons also play a crucial role in promoting programmed cell death and checking cell growth [10]. Owing to these benefits, alpha interferon has been used in various cancers, such as renal cancer and melanomas [11].

Moreover, it was also approved for solid and hematologic tumors. It has been reported that interferon levels significantly declined in colon cancer patients, indicating that colon cancer patients are deficient in interferon signaling [9]. This generates a hypothesis that exogenous interferon delivery might help reduce the tumor cells. In this *in vitro* research, the effect of interferon on colon cancer cells cultured *in vitro* was studied and compared with Cisplatin.

## Material and methods

### Materials

Human SW 480 cell line was obtained from the cancer research unit in Babylon medical college. All plasticware, including tissue culture plates, flask falcons, pipettes, along with phosphate-buffered saline, RPMI-1630 culture media, trypsin, and other reagents, was bought from local scientific companies. Cisplatin was obtained in a vial and purchased from a local pharmacy. Interferon-alpha was prepared in the Science Laboratories of the University of Belarus (Belarusian State).

### Method

The SW 480 cell line was grown in the cancer research laboratory and seeded on 96 micro-culture plates until it became an 80% monolayer. Then it was exposed to interferon-alpha in serial dilution concentrations starting from 2.5 μg to 0.057 μg in triplicates. Three replicates were left as a control group without exposure to interferon. After 24 hours, the media and reagent were pipetted and discarded. The plate was stained with crystal violet (CV) stain as per CV staining protocols. An ELISA reader was used to read the optic density of the wells. The same experiment was repeated with cisplatin chemotherapy starting with 100 μg to 3.125 μg in six serial dilution concentrations. Also, after 24 hours, the result was read with an ELISA reader at 570 wavelengths. The exact process was repeated for other dilutions left for 48 hours and 72 hours.

### Statistical analysis

To statistically analyze the results, we depend on the ability of interferon to inhibit the growth of colon cancer cells compared to the known effect of Cisplatin. The IR%, *i.e.*, percentage of inhibitory rate, was calculated according to the equation:



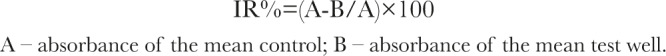



This equation calculates the inhibition ability of different interferon concentrations. The results are then compared with the inhibition coefficient of Cisplatin for the same periods. The corresponding concentrations according to the dilutions start from the highest concentration (2.5 μg/ml in case of alpha interferon and 100 μg/ml in case of Cisplatin) to the lowest concentration (0.057 μg/ml in case of alpha interferon and 3.25 μg/ml in case of Cisplatin). It is worth mentioning that interferon concentrations differ from the concentrations of Cisplatin because they are two different substances. Finally, the effect factor was calculated for the six concentrations, using the same control rate at each time within the same table.

## Results

There were six concentrations of alpha interferon (2.5 μg/ml, 1.25 μg/ml, 0.562 μg/ml, 0.286 μg/ml, 0.143 μg/ml, and 0.057 μg/ml) along with a control group. These values were compared with the six concentrations of Cisplatin (100 μg/ml, 50 μg/ml, 25 μg/ml, 6.25 μg/ml, and 3.125) along with a control group. These concentrations were placed for 24 hours, 48 hours, and 72 hours separately for analysis. All tests were done in triplicates.

### Percentage inhibition

Percentage inhibition represents the level of inhibition of cell growth in the test sample. After obtaining the results from the ELISA plate reader, here are the results of the analysis:

### Rate of cell growth inhibition at 24 hours

After 24 hours, the overall inhibition rate of interferon was observed to be higher than Cisplatin. Consequently, alpha-interferon showed more activity at reducing the cancer cell burden. Of all the concentrations, 1.25 μg/ml showed the highest percentage inhibition rate, *i.e.*, 91.24%. Tables 1 and 2 show a detailed comparison between interferon and cisplatin inhibition levels at different concentrations.

**Table 1.** Interferon inhibition levels at different concentrations after 24 hr.

**Concentration of interferon (μg/ml)**	**Mean absorption of test wells**	**Mean absorption of control well**	**%IR**
**2.5**	0.066	0.719	90.82
**1.25**	0.063	0.719	91.24
**0.562**	0.107	0.719	85.11
**0.286**	0.307	0.719	57.30
**0.143**	0.537	0.719	25.31
**0.057**	0.624	0.719	13.21

**Table 2.** Cisplatin inhibition levels at different concentrations after 24 hr.

Concentrations of Cisplatin (μg/ml)	Mean absorption of test wells	Mean absorption of control well	%IR
**100**	0.484	0.794	39.04
**50**	0.592	0.794	25.44
**25**	0.724	0.794	8.81
**12.5**	0.728	0.794	8.31
**6.25**	0.76	0.794	4.28
**3.125**	0.781	0.794	1.64

### Rate of cell growth inhibition at 48 hours

After 48 hours, results were compared with interferon for a higher percentage inhibitory ratio. It was observed that the higher the concentration, the higher the inhibitory ratio obtained with 2.5 μg/ml showing the maximum inhibition, *i.e.*, 93.830% (Tables 3 and 4).

**Table 3.** Interferon inhibition levels at different concentrations after 48 hr.

**Concentrations of interferon (μg/ml)**	**Mean absorption of test wells**	**Mean absorption of control well**	**%IR**
**2.5**	0.058	0.94	93.830
**1.25**	0.059	0.94	93.723
**0.562**	0.096	0.94	89.787
**0.286**	0.021	0.94	87.766
**0.143**	0.121	0.94	87.128
**0.057**	0.601	0.94	36.064

**Table 4.** Cisplatin inhibition levels at different concentrations after 48 hr.

**Concentrations of Cisplatin (μg/ml)**	**Mean absorption of test wells**	**Mean absorption of control well**	**%IR**
**100**	0.25	1.066	76.548
**50**	0.383	1.066	64.071
**25**	0.331	1.066	68.949
**12.5**	0.399	1.066	62.570
**6.25**	0.371	1.066	65.197
**3.125**	0.755	1.066	29.174

### Rate of cell growth inhibition at 72 hours

After 72 hours, comparable results with the previous finding at 24 and 48 hours were obtained. Alpha interferon showed maximum inhibition at 72-hour exposure with values above 90% and 0.286 μg/ml with a maximum inhibition of 93.485% (Tables 5 and 6). However, it was observed that Cisplatin induced proliferation instead of inhibition at a lower concentration as negative results of percentage inhibition ratio were obtained.

**Table 5.** Interferon inhibition levels at different concentrations after 72 hr.

**Concentrations of interferon (μg/ml)**	**Mean absorption of test wells**	**Mean absorption of control well**	**%IR**
**2.5**	0.078	0.967	91.934
**1.25**	0.065	0.967	93.278
**0.562**	0.065	0.967	93.278
**0.286**	0.063	0.967	93.485
**0.143**	0.064	0.967	93.382
**0.057**	0.073	0.967	92.451

**Table 6.** Cisplatin inhibition levels at different concentrations after 48 hr.

**Concentrations of Cisplatin (μg/ml)**	**Mean absorption of test wells**	**Mean absorption of control well**	**%IR**
**100**	0.362	0.93	61.075
**50**	0.488	0.93	47.527
**25**	0.436	0.93	53.118
**12.5**	0.572	0.93	38.495
**6.25**	1.054	0.93	-13.333
**3.125**	1.051	0.93	-13.011

We used analysis of variance (ANOVA) to compare the means. According to ANOVA results, after 24 hours, the alpha interferon at concentrations 0.057 μg/ml, 0.143 μg/ml and 2.5 μg/ml showed statistically significant inhibition, with F_2-6_=26.536=0.001, F_2-6_=80.295=0.00 and F_2-6_=5.44=0.45, respectively (Table 7).

**Table 7.** Interferon inhibition levels at different concentrations.

	**F**	**Sig**
**Control**	13.705	0.006
**0.057**	26.536	0.001
**0.143**	80.295	0.000
**0.286**	3.653	0.092
**0.562**	0.623	0.568
**1.25**	0.519	0.619
**2.50**	5.444	0.045

When the statistical analysis was compared within the group at different hours, it was found that maximum inhibition was observed at concentration 0.143 μg/ml when exposed for 48 to 72 hours. In comparison, maximum inhibition was observed with a 25 μg/ml concentration of Cisplatin. The summary of the analysis is shown in Tables 8 and 9. When we compared the activity of Interferon and Cisplatin, significant results were observed at concentrations 50 μg/ml, 25 μg/ml, 12.5 μg/ml, and 3.12 μg/ml.

**Table 8.** Effects of Interferon on the biomarkers for cancer suppression (ANOVA).

**Dependent Variable**	**(I)**	**(J)**	**Mean Difference (I-J)**	**Sig.**
**control**	interferon 24 h	interferon 48 h	-0.2217(*)	.013
		interferon 72 h	-0.2487(*)	.007
	interferon 48 h	interferon 24 h	0.2217(*)	.013
		interferon 72 h	-2.7000E-02	.866
	**interferon 72 h**	interferon 24 h	0.2487(*)	.007
		interferon 48 h	2.700E-02	.866
**0.057**	interferon 24 h	interferon 48 h	2.267E-02	.962
		interferon 72 h	0.5510(*)	.002
	**interferon 48 h**	interferon 24 h	-2.2667E-02	.962
		interferon 72 h	0.5283(*)	.002
	**interferon 72 h**	interferon 24 h	-0.5510(*)	.002
		interferon 48 h	-0.5283(*)	.002
**0.143**	interferon 24 h	interferon 48 h	0.4063(*)	.000
		interferon 72 h	0.4630(*)	.000
	**interferon 48 h**	interferon 24 h	-0.4063(*)	.000
		interferon 72 h	5.667E-02	.389
	**interferon 72 h**	interferon 24 h	-0.4630(*)	.000
		interferon 48 h	-5.6667E-02	.389
**0.286**	interferon 24 h	interferon 48 h	0.2360	.131
		interferon 72 h	0.2437	.119
	**interferon 48 h**	interferon 24 h	-0.2360	.131
		interferon 72 h	7.667E-03	.997
	**interferon 72 h**	interferon 24 h	-0.2437	.119
		interferon 48 h	-7.6667E-03	.997
**0.562**	interferon 24 h	interferon 48 h	1.033E-02	.962
		interferon 72 h	4.167E-02	.564
	**interferon 48 h**	interferon 24 h	-1.0333E-02	.962
		interferon 72 h	3.133E-02	.713
	**interferon 72 h**	interferon 24 h	-4.1667E-02	.564
		interferon 48 h	-3.1333E-02	.713
**1.25**	interferon 24 h	interferon 48 h	5.667E-03	.730
		interferon 72 h	-1.3333E-03	.982
	**interferon 48 h**	interferon 24 h	-5.6667E-03	.730
		interferon 72 h	-7.0000E-03	.626
	**interferon 72 h**	interferon 24 h	1.333E-03	.982
		interferon 48 h	7.000E-03	.626
**2.5**	interferon 24 h	interferon 48 h	1.000E-03	.987
		interferon 72 h	-1.8000E-02	.072
	**interferon 48 h**	interferon 24 h	-1.0000E-03	.987
		interferon 72 h	-1.9000E-02	.059
	**interferon 72 h**	interferon 24 h	1.800E-02	.072
		interferon 48 h	1.900E-02	.059

**Table 9.** Effects of Cisplatin on the biomarkers for cancer suppression (ANOVA).

**Dependent Variable**	**(I) grouping**	**(J) grouping**	**Mean Difference (I-J)**	**Sig.**
**control**	cisplatin 24 h	cisplatin 48 h	-.2720(*)	0.003
		cisplatin 72 h	-0.1360	0.058
	cisplatin 48 h	cisplatin 24 h	0.2720(*)	0.003
		cisplatin 72 h	0.1360	.058
	cisplatin 72 h	cisplatin 24 h	0.1360	0.058
		cisplatin 48 h	-0.1360	0.058
**3.25**	cisplatin 24 h	cisplatin 48 h	2.633E-02	0.977
		cisplatin 72 h	-0.2707	0.170
	**cisplatin 48 h**	cisplatin 24 h	-2.6333E-02	.977
		cisplatin 72 h	-0.2970	0.131
	cisplatin 72 h	cisplatin 24 h	0.2707	0.170
		cisplatin 48 h	0.2970	0.131
**6.5**	cisplatin 24 h	cisplatin 48 h	0.4010(*)	0.003
		cisplatin 72 h	-0.2943(*)	0.012
	cisplatin 48 h	cisplatin 24 h	-0.4010(*)	0.003
		cisplatin 72 h	-0.6953(*)	0.000
	cisplatin 72 h	cisplatin 24 h	0.2943(*)	0.012
		cisplatin 48 h	0.6953(*)	0.000
**12.5**	cisplatin 24 h	cisplatin 48 h	0.3290(*)	0.008
		cisplatin 72 h	0.1560	0.150
	cisplatin 48 h	cisplatin 24 h	-0.3290(*)	0.008
		cisplatin 72 h	-0.1730	0.110
	cisplatin 72 h	cisplatin 24 h	-0.1560	0.150
		cisplatin 48 h	0.1730	0.110
**25**	cisplatin 24 h	cisplatin 48 h	0.3933(*)	0.000
		cisplatin 72 h	0.2887(*)	0.000
	cisplatin 48 h	cisplatin 24 h	-0.3933(*)	0.000
		cisplatin 72 h	-0.1047	0.056
	cisplatin 72 h	cisplatin 24 h	-0.2887(*)	0.000
		cisplatin 48 h	0.1047	0.056
**50**	cisplatin 24 h	cisplatin 48 h	0.2090	0.056
		cisplatin 72 h	0.1040	0.364
	cisplatin 48 h	cisplatin 24 h	-0.2090	0.056
		cisplatin 72 h	-0.1050	0.358
	cisplatin 72 h	cisplatin 24 h	-0.1040	0.364
		cisplatin 48 h	0.1050	0.358
**100**	cisplatin 24 h	cisplatin 48 h	0.2350(*)	0.001
		cisplatin 72 h	0.1330(*)	0.014
	cisplatin 48 h	cisplatin 24 h	-0.2350(*)	0.001
		cisplatin 72 h	-0.1020(*)	0.043
	cisplatin 72 h	cisplatin 24 h	-0.1330(*)	0.014
		cisplatin 48 h	0.1020(*)	0.043

## Discussion

### Percentage inhibition

The results of percentage inhibition indicate an excellent anti-cancer activity of alpha interferon in the colon cancer cell line. In general, all concentrations of alpha interferon showed significant activity. The order of activity was observed to be as 2.5>1.25>0.562>0.286>0.143>0.057 μg/ml at 24 hours, 0.286>2.5>1.25>0.562>0.143>0.057 μg/ml at 48 hours and 0.285>0.143>1.25, 0.562>0.057>2.5 μg/ml at 72 hours.

In comparison to Cisplatin, alpha-interferon was more potent in all cases since the percentage inhibition of Cisplatin was below 80%. Furthermore, it was observed that Cisplatin induced proliferation at concentrations 6.25 μg/ml and 3.125 μg/ml after 72 hours of exposure. All of the results were done thrice and summarized in the tables.

ANOVA analysis was carried out to investigate the significant differences between the means. It showed that the alpha interferon at concentrations 0.057 μg/ml, 0.143 μg/ml, and 2.5 μg/ml were statistically significant for the cancer cell inhibition, with F_2-6_=26.536=0.001, F_2-6_=80.295=0.00, and F_2-6_=5.44=0.45, respectively.

The statistical analysis of interferon was compared within the group at different hours, and it was observed that maximum inhibition occurred at concentration 0.143 μg/ml when exposed for 48 to 72 hours.

In comparison, maximum inhibition was observed with a 25 μg/ml concentration of Cisplatin. Hence the ANOVA results suggest that the percentage of inhibition of alpha interferon was significant. These findings are consistent with previous findings that indicate that alpha-interferon suppresses tumor growth in colon cancer [12–16]. The in vivo anticancer activity of alpha interferon is proven in other cancers. Still, current *in vitro* findings strongly support further analysis, particularly in vivo, to confirm its activity against colon cancer [17–19].

## Conclusion

Alpha interferon, well-known for its viral suppression activity, is gaining clinical interest for its cancer suppression activity. This *in vitro* analysis demonstrated that alpha interferons have anti-cancer activity against colon cancer cell lines and higher activity than Cisplatin.

## Acknowledgments

### Conflict of interest

The authors declare no conflict of interest.

### Ethical approval

This study was approved by the Scientific and Ethical Committees of Al-Bayan University. This study was registered in the Department of Scientific Affairs at the Al-Bayan University (protocol 167, 2017).

### Funding

This study was co-funded by the authors and Al-Bayan University.

### Personal thanks

This work was supported by the College of Pharmacy, Al-Bayan University. The authors thank the National Center of Cancer Research in Baghdad for their cooperation and support.

### Authorship

AM contributed to conceptualizing the study, data collection, and collecting samples, media, and other materials. IA contributed to writing the original draft and data analysis. HH contributed to the methodology of the study and to editing the manuscript.
